# Supplementary material for: The effects of genital myiasis on the diversity of the vaginal microbiota in female Bactrian camels
Source: BMC Vet Res. 2022 Mar 5;18:87. doi: 10.1186/s12917-022-03189-5 (PMC8897907; doi:10.1186/s12917-022-03189-5)
Supplement: Supplementary file 5 — Additional file 5. [file 12917_2022_3189_MOESM5_ESM.zip › MPL201709200_16s_yy/Treat1/css/js_css/jsPDF-master.zip!/jsPDF-master/docs/plugins_addimage.js.html]

plugins/addimage.js - Documentation


## Home

### Classes

- jsPDF

### Global

- addFont
- addHTML
- addMetadata
- addPage
- autoPrint
- CapJoinStyles
- circle
- ellipse
- getFontList
- lines
- lstext
- output
- rect
- roundedRect
- save
- setDisplayMode
- setDrawColor
- setFillColor
- setFont
- setFontSize
- setFontStyle
- setLineCap
- setLineJoin
- setLineWidth
- setPage
- setProperties
- setTextColor
- text
- triangle

# plugins/addimage.js

```
/** @preserve
 * jsPDF addImage plugin
 * Copyright (c) 2012 Jason Siefken, https://github.com/siefkenj/
 *               2013 Chris Dowling, https://github.com/gingerchris
 *               2013 Trinh Ho, https://github.com/ineedfat
 *               2013 Edwin Alejandro Perez, https://github.com/eaparango
 *               2013 Norah Smith, https://github.com/burnburnrocket
 *               2014 Diego Casorran, https://github.com/diegocr
 *               2014 James Robb, https://github.com/jamesbrobb
 *
 * Permission is hereby granted, free of charge, to any person obtaining
 * a copy of this software and associated documentation files (the
 * "Software"), to deal in the Software without restriction, including
 * without limitation the rights to use, copy, modify, merge, publish,
 * distribute, sublicense, and/or sell copies of the Software, and to
 * permit persons to whom the Software is furnished to do so, subject to
 * the following conditions:
 *
 * The above copyright notice and this permission notice shall be
 * included in all copies or substantial portions of the Software.
 *
 * THE SOFTWARE IS PROVIDED "AS IS", WITHOUT WARRANTY OF ANY KIND,
 * EXPRESS OR IMPLIED, INCLUDING BUT NOT LIMITED TO THE WARRANTIES OF
 * MERCHANTABILITY, FITNESS FOR A PARTICULAR PURPOSE AND
 * NONINFRINGEMENT. IN NO EVENT SHALL THE AUTHORS OR COPYRIGHT HOLDERS BE
 * LIABLE FOR ANY CLAIM, DAMAGES OR OTHER LIABILITY, WHETHER IN AN ACTION
 * OF CONTRACT, TORT OR OTHERWISE, ARISING FROM, OUT OF OR IN CONNECTION
 * WITH THE SOFTWARE OR THE USE OR OTHER DEALINGS IN THE SOFTWARE.
 */

;(function(jsPDFAPI) {
	'use strict'

	var namespace = 'addImage_',
		supported_image_types = ['jpeg', 'jpg', 'png'];

	// Image functionality ported from pdf.js
	var putImage = function(img) {

		var objectNumber = this.internal.newObject()
		, out = this.internal.write
		, putStream = this.internal.putStream

		img['n'] = objectNumber

		out('<</Type /XObject')
		out('/Subtype /Image')
		out('/Width ' + img['w'])
		out('/Height ' + img['h'])
		if (img['cs'] === this.color_spaces.INDEXED) {
			out('/ColorSpace [/Indexed /DeviceRGB '
					// if an indexed png defines more than one colour with transparency, we've created a smask
					+ (img['pal'].length / 3 - 1) + ' ' + ('smask' in img ? objectNumber + 2 : objectNumber + 1)
					+ ' 0 R]');
		} else {
			out('/ColorSpace /' + img['cs']);
			if (img['cs'] === this.color_spaces.DEVICE_CMYK) {
				out('/Decode [1 0 1 0 1 0 1 0]');
			}
		}
		out('/BitsPerComponent ' + img['bpc']);
		if ('f' in img) {
			out('/Filter /' + img['f']);
		}
		if ('dp' in img) {
			out('/DecodeParms <<' + img['dp'] + '>>');
		}
		if ('trns' in img && img['trns'].constructor == Array) {
			var trns = '',
				i = 0,
				len = img['trns'].length;
			for (; i < len; i++)
				trns += (img['trns'][i] + ' ' + img['trns'][i] + ' ');
			out('/Mask [' + trns + ']');
		}
		if ('smask' in img) {
			out('/SMask ' + (objectNumber + 1) + ' 0 R');
		}
		out('/Length ' + img['data'].length + '>>');

		putStream(img['data']);

		out('endobj');

		// Soft mask
		if ('smask' in img) {
			var dp = '/Predictor '+ img['p'] +' /Colors 1 /BitsPerComponent ' + img['bpc'] + ' /Columns ' + img['w'];
			var smask = {'w': img['w'], 'h': img['h'], 'cs': 'DeviceGray', 'bpc': img['bpc'], 'dp': dp, 'data': img['smask']};
			if ('f' in img)
				smask.f = img['f'];
			putImage.call(this, smask);
		}

	    //Palette
		if (img['cs'] === this.color_spaces.INDEXED) {

			this.internal.newObject();
			//out('<< /Filter / ' + img['f'] +' /Length ' + img['pal'].length + '>>');
			//putStream(zlib.compress(img['pal']));
			out('<< /Length ' + img['pal'].length + '>>');
			putStream(this.arrayBufferToBinaryString(new Uint8Array(img['pal'])));
			out('endobj');
		}
	}
	, putResourcesCallback = function() {
		var images = this.internal.collections[namespace + 'images']
		for ( var i in images ) {
			putImage.call(this, images[i])
		}
	}
	, putXObjectsDictCallback = function(){
		var images = this.internal.collections[namespace + 'images']
		, out = this.internal.write
		, image
		for (var i in images) {
			image = images[i]
			out(
				'/I' + image['i']
				, image['n']
				, '0'
				, 'R'
			)
		}
	}
	, checkCompressValue = function(value) {
		if(value && typeof value === 'string')
			value = value.toUpperCase();
		return value in jsPDFAPI.image_compression ? value : jsPDFAPI.image_compression.NONE;
	}
	, getImages = function() {
		var images = this.internal.collections[namespace + 'images'];
		//first run, so initialise stuff
		if(!images) {
			this.internal.collections[namespace + 'images'] = images = {};
			this.internal.events.subscribe('putResources', putResourcesCallback);
			this.internal.events.subscribe('putXobjectDict', putXObjectsDictCallback);
		}

		return images;
	}
	, getImageIndex = function(images) {
		var imageIndex = 0;

		if (images){
			// this is NOT the first time this method is ran on this instance of jsPDF object.
			imageIndex = Object.keys ?
			Object.keys(images).length :
			(function(o){
				var i = 0
				for (var e in o){if(o.hasOwnProperty(e)){ i++ }}
				return i
			})(images)
		}

		return imageIndex;
	}
	, notDefined = function(value) {
		return typeof value === 'undefined' || value === null;
	}
	, generateAliasFromData = function(data) {
		return typeof data === 'string' && jsPDFAPI.sHashCode(data);
	}
	, doesNotSupportImageType = function(type) {
		return supported_image_types.indexOf(type) === -1;
	}
	, processMethodNotEnabled = function(type) {
		return typeof jsPDFAPI['process' + type.toUpperCase()] !== 'function';
	}
	, isDOMElement = function(object) {
		return typeof object === 'object' && object.nodeType === 1;
	}
	, createDataURIFromElement = function(element, format, angle) {

		//if element is an image which uses data url definition, just return the dataurl
		if (element.nodeName === 'IMG' && element.hasAttribute('src')) {
			var src = ''+element.getAttribute('src');
			if (!angle && src.indexOf('data:image/') === 0) return src;

			// only if the user doesn't care about a format
			if (!format && /\.png(?:[?#].*)?$/i.test(src)) format = 'png';
		}

		if(element.nodeName === 'CANVAS') {
			var canvas = element;
		} else {
			var canvas = document.createElement('canvas');
			canvas.width = element.clientWidth || element.width;
			canvas.height = element.clientHeight || element.height;

			var ctx = canvas.getContext('2d');
			if (!ctx) {
				throw ('addImage requires canvas to be supported by browser.');
			}
			if (angle) {
				var x, y, b, c, s, w, h, to_radians = Math.PI/180, angleInRadians;

				if (typeof angle === 'object') {
					x = angle.x;
					y = angle.y;
					b = angle.bg;
					angle = angle.angle;
				}
				angleInRadians = angle*to_radians;
				c = Math.abs(Math.cos(angleInRadians));
				s = Math.abs(Math.sin(angleInRadians));
				w = canvas.width;
				h = canvas.height;
				canvas.width = h * s + w * c;
				canvas.height = h * c + w * s;

				if (isNaN(x)) x = canvas.width / 2;
				if (isNaN(y)) y = canvas.height / 2;

				ctx.clearRect(0,0,canvas.width, canvas.height);
				ctx.fillStyle = b || 'white';
				ctx.fillRect(0, 0, canvas.width, canvas.height);
				ctx.save();
				ctx.translate(x, y);
				ctx.rotate(angleInRadians);
				ctx.drawImage(element, -(w/2), -(h/2));
				ctx.rotate(-angleInRadians);
				ctx.translate(-x, -y);
				ctx.restore();
			} else {
				ctx.drawImage(element, 0, 0, canvas.width, canvas.height);
			}
		}
		return canvas.toDataURL((''+format).toLowerCase() == 'png' ? 'image/png' : 'image/jpeg');
	}
	,checkImagesForAlias = function(alias, images) {
		var cached_info;
		if(images) {
			for(var e in images) {
				if(alias === images[e].alias) {
					cached_info = images[e];
					break;
				}
			}
		}
		return cached_info;
	}
	,determineWidthAndHeight = function(w, h, info) {
		if (!w && !h) {
			w = -96;
			h = -96;
		}
		if (w < 0) {
			w = (-1) * info['w'] * 72 / w / this.internal.scaleFactor;
		}
		if (h < 0) {
			h = (-1) * info['h'] * 72 / h / this.internal.scaleFactor;
		}
		if (w === 0) {
			w = h * info['w'] / info['h'];
		}
		if (h === 0) {
			h = w * info['h'] / info['w'];
		}

		return [w, h];
	}
	, writeImageToPDF = function(x, y, w, h, info, index, images) {
		var dims = determineWidthAndHeight.call(this, w, h, info),
			coord = this.internal.getCoordinateString,
			vcoord = this.internal.getVerticalCoordinateString;

		w = dims[0];
		h = dims[1];

		images[index] = info;

		this.internal.write(
			'q'
			, coord(w)
			, '0 0'
			, coord(h) // TODO: check if this should be shifted by vcoord
			, coord(x)
			, vcoord(y + h)
			, 'cm /I'+info['i']
			, 'Do Q'
		)
	};

	/**
	 * COLOR SPACES
	 */
	jsPDFAPI.color_spaces = {
		DEVICE_RGB:'DeviceRGB',
		DEVICE_GRAY:'DeviceGray',
		DEVICE_CMYK:'DeviceCMYK',
		CAL_GREY:'CalGray',
		CAL_RGB:'CalRGB',
		LAB:'Lab',
		ICC_BASED:'ICCBased',
		INDEXED:'Indexed',
		PATTERN:'Pattern',
		SEPARATION:'Separation',
		DEVICE_N:'DeviceN'
	};

	/**
	 * DECODE METHODS
	 */
	jsPDFAPI.decode = {
		DCT_DECODE:'DCTDecode',
		FLATE_DECODE:'FlateDecode',
		LZW_DECODE:'LZWDecode',
		JPX_DECODE:'JPXDecode',
		JBIG2_DECODE:'JBIG2Decode',
		ASCII85_DECODE:'ASCII85Decode',
		ASCII_HEX_DECODE:'ASCIIHexDecode',
		RUN_LENGTH_DECODE:'RunLengthDecode',
		CCITT_FAX_DECODE:'CCITTFaxDecode'
	};

	/**
	 * IMAGE COMPRESSION TYPES
	 */
	jsPDFAPI.image_compression = {
		NONE: 'NONE',
		FAST: 'FAST',
		MEDIUM: 'MEDIUM',
		SLOW: 'SLOW'
	};

	jsPDFAPI.sHashCode = function(str) {
		return Array.prototype.reduce && str.split("").reduce(function(a,b){a=((a<<5)-a)+b.charCodeAt(0);return a&a},0);
	};

	jsPDFAPI.isString = function(object) {
		return typeof object === 'string';
	};

	/**
	 * Strips out and returns info from a valid base64 data URI
	 * @param {String[dataURI]} a valid data URI of format 'data:[<MIME-type>][;base64],<data>'
	 * @returns an Array containing the following
	 * [0] the complete data URI
	 * [1] <MIME-type>
	 * [2] format - the second part of the mime-type i.e 'png' in 'image/png'
	 * [4] <data>
	 */
	jsPDFAPI.extractInfoFromBase64DataURI = function(dataURI) {
		return /^data:([\w]+?\/([\w]+?));base64,(.+?)$/g.exec(dataURI);
	};

	/**
	 * Check to see if ArrayBuffer is supported
	 */
	jsPDFAPI.supportsArrayBuffer = function() {
		return typeof ArrayBuffer !== 'undefined' && typeof Uint8Array !== 'undefined';
	};

	/**
	 * Tests supplied object to determine if ArrayBuffer
	 * @param {Object[object]}
	 */
	jsPDFAPI.isArrayBuffer = function(object) {
		if(!this.supportsArrayBuffer())
	        return false;
		return object instanceof ArrayBuffer;
	};

	/**
	 * Tests supplied object to determine if it implements the ArrayBufferView (TypedArray) interface
	 * @param {Object[object]}
	 */
	jsPDFAPI.isArrayBufferView = function(object) {
		if(!this.supportsArrayBuffer())
	        return false;
		if(typeof Uint32Array === 'undefined')
			return false;
		return (object instanceof Int8Array ||
				object instanceof Uint8Array ||
				(typeof Uint8ClampedArray !== 'undefined' && object instanceof Uint8ClampedArray) ||
				object instanceof Int16Array ||
				object instanceof Uint16Array ||
				object instanceof Int32Array ||
				object instanceof Uint32Array ||
				object instanceof Float32Array ||
				object instanceof Float64Array );
	};

	/**
	 * Exactly what it says on the tin
	 */
	jsPDFAPI.binaryStringToUint8Array = function(binary_string) {
		/*
		 * not sure how efficient this will be will bigger files. Is there a native method?
		 */
		var len = binary_string.length;
	    var bytes = new Uint8Array( len );
	    for (var i = 0; i < len; i++) {
	        bytes[i] = binary_string.charCodeAt(i);
	    }
	    return bytes;
	};

	/**
	 * @see this discussion
	 * http://stackoverflow.com/questions/6965107/converting-between-strings-and-arraybuffers
	 *
	 * As stated, i imagine the method below is highly inefficent for large files.
	 *
	 * Also of note from Mozilla,
	 *
	 * "However, this is slow and error-prone, due to the need for multiple conversions (especially if the binary data is not actually byte-format data, but, for example, 32-bit integers or floats)."
	 *
	 * https://developer.mozilla.org/en-US/Add-ons/Code_snippets/StringView
	 *
	 * Although i'm strugglig to see how StringView solves this issue? Doesn't appear to be a direct method for conversion?
	 *
	 * Async method using Blob and FileReader could be best, but i'm not sure how to fit it into the flow?
	 */
	jsPDFAPI.arrayBufferToBinaryString = function(buffer) {
		/*if('TextDecoder' in window){
			var decoder = new TextDecoder('ascii');
			return decoder.decode(buffer);
		}*/

		if(this.isArrayBuffer(buffer))
			buffer = new Uint8Array(buffer);

	    var binary_string = '';
	    var len = buffer.byteLength;
	    for (var i = 0; i < len; i++) {
	        binary_string += String.fromCharCode(buffer[i]);
	    }
	    return binary_string;
	    /*
	     * Another solution is the method below - convert array buffer straight to base64 and then use atob
	     */
		//return atob(this.arrayBufferToBase64(buffer));
	};

	/**
	 * Converts an ArrayBuffer directly to base64
	 *
	 * Taken from here
	 *
	 * http://jsperf.com/encoding-xhr-image-data/31
	 *
	 * Need to test if this is a better solution for larger files
	 *
	 */
	jsPDFAPI.arrayBufferToBase64 = function(arrayBuffer) {
		var base64    = ''
		var encodings = 'ABCDEFGHIJKLMNOPQRSTUVWXYZabcdefghijklmnopqrstuvwxyz0123456789+/'

		var bytes         = new Uint8Array(arrayBuffer)
		var byteLength    = bytes.byteLength
		var byteRemainder = byteLength % 3
		var mainLength    = byteLength - byteRemainder

		var a, b, c, d
		var chunk

		// Main loop deals with bytes in chunks of 3
		for (var i = 0; i < mainLength; i = i + 3) {
			// Combine the three bytes into a single integer
			chunk = (bytes[i] << 16) | (bytes[i + 1] << 8) | bytes[i + 2]

			// Use bitmasks to extract 6-bit segments from the triplet
			a = (chunk & 16515072) >> 18 // 16515072 = (2^6 - 1) << 18
			b = (chunk & 258048)   >> 12 // 258048   = (2^6 - 1) << 12
			c = (chunk & 4032)     >>  6 // 4032     = (2^6 - 1) << 6
			d = chunk & 63               // 63       = 2^6 - 1

			// Convert the raw binary segments to the appropriate ASCII encoding
			base64 += encodings[a] + encodings[b] + encodings[c] + encodings[d]
		}

		// Deal with the remaining bytes and padding
		if (byteRemainder == 1) {
			chunk = bytes[mainLength]

			a = (chunk & 252) >> 2 // 252 = (2^6 - 1) << 2

			// Set the 4 least significant bits to zero
			b = (chunk & 3)   << 4 // 3   = 2^2 - 1

			base64 += encodings[a] + encodings[b] + '=='
		} else if (byteRemainder == 2) {
			chunk = (bytes[mainLength] << 8) | bytes[mainLength + 1]

			a = (chunk & 64512) >> 10 // 64512 = (2^6 - 1) << 10
			b = (chunk & 1008)  >>  4 // 1008  = (2^6 - 1) << 4

			// Set the 2 least significant bits to zero
			c = (chunk & 15)    <<  2 // 15    = 2^4 - 1

			base64 += encodings[a] + encodings[b] + encodings[c] + '='
		}

		return base64
	};

	jsPDFAPI.createImageInfo = function(data, wd, ht, cs, bpc, f, imageIndex, alias, dp, trns, pal, smask, p) {
		var info = {
				alias:alias,
				w : wd,
				h : ht,
				cs : cs,
				bpc : bpc,
				i : imageIndex,
				data : data
				// n: objectNumber will be added by putImage code
			};

		if(f) info.f = f;
		if(dp) info.dp = dp;
		if(trns) info.trns = trns;
		if(pal) info.pal = pal;
		if(smask) info.smask = smask;
		if(p) info.p = p;// predictor parameter for PNG compression

		return info;
	};

	jsPDFAPI.addImage = function(imageData, format, x, y, w, h, alias, compression, rotation) {
		'use strict'

		if(typeof format !== 'string') {
			var tmp = h;
			h = w;
			w = y;
			y = x;
			x = format;
			format = tmp;
		}

		if (typeof imageData === 'object' && !isDOMElement(imageData) && "imageData" in imageData) {
			var options = imageData;

			imageData = options.imageData;
			format = options.format || format;
			x = options.x || x || 0;
			y = options.y || y || 0;
			w = options.w || w;
			h = options.h || h;
			alias = options.alias || alias;
			compression = options.compression || compression;
			rotation = options.rotation || options.angle || rotation;
		}

		if (isNaN(x) || isNaN(y))
		{
			console.error('jsPDF.addImage: Invalid coordinates', arguments);
			throw new Error('Invalid coordinates passed to jsPDF.addImage');
		}

		var images = getImages.call(this), info;

		if (!(info = checkImagesForAlias(imageData, images))) {
			var dataAsBinaryString;

			if(isDOMElement(imageData))
				imageData = createDataURIFromElement(imageData, format, rotation);

			if(notDefined(alias))
				alias = generateAliasFromData(imageData);

			if (!(info = checkImagesForAlias(alias, images))) {

				if(this.isString(imageData)) {

					var base64Info = this.extractInfoFromBase64DataURI(imageData);

					if(base64Info) {

						format = base64Info[2];
						imageData = atob(base64Info[3]);//convert to binary string

					} else {

						if (imageData.charCodeAt(0) === 0x89 &&
							imageData.charCodeAt(1) === 0x50 &&
							imageData.charCodeAt(2) === 0x4e &&
							imageData.charCodeAt(3) === 0x47  )  format = 'png';
					}
				}
				format = (format || 'JPEG').toLowerCase();

				if(doesNotSupportImageType(format))
					throw new Error('addImage currently only supports formats ' + supported_image_types + ', not \''+format+'\'');

				if(processMethodNotEnabled(format))
					throw new Error('please ensure that the plugin for \''+format+'\' support is added');

				/**
				 * need to test if it's more efficient to convert all binary strings
				 * to TypedArray - or should we just leave and process as string?
				 */
				if(this.supportsArrayBuffer()) {
					// no need to convert if imageData is already uint8array
					if(!(imageData instanceof Uint8Array)){
						dataAsBinaryString = imageData;
						imageData = this.binaryStringToUint8Array(imageData);
					}
				}

				info = this['process' + format.toUpperCase()](
					imageData,
					getImageIndex(images),
					alias,
					checkCompressValue(compression),
					dataAsBinaryString
				);

				if(!info)
					throw new Error('An unkwown error occurred whilst processing the image');
			}
		}

		writeImageToPDF.call(this, x, y, w, h, info, info.i, images);

		return this
	};

	/**
	 * JPEG SUPPORT
	 **/

	//takes a string imgData containing the raw bytes of
	//a jpeg image and returns [width, height]
	//Algorithm from: http://www.64lines.com/jpeg-width-height
	var getJpegSize = function(imgData) {
		'use strict'
		var width, height, numcomponents;
		// Verify we have a valid jpeg header 0xff,0xd8,0xff,0xe0,?,?,'J','F','I','F',0x00
		if (!imgData.charCodeAt(0) === 0xff ||
			!imgData.charCodeAt(1) === 0xd8 ||
			!imgData.charCodeAt(2) === 0xff ||
			!imgData.charCodeAt(3) === 0xe0 ||
			!imgData.charCodeAt(6) === 'J'.charCodeAt(0) ||
			!imgData.charCodeAt(7) === 'F'.charCodeAt(0) ||
			!imgData.charCodeAt(8) === 'I'.charCodeAt(0) ||
			!imgData.charCodeAt(9) === 'F'.charCodeAt(0) ||
			!imgData.charCodeAt(10) === 0x00) {
				throw new Error('getJpegSize requires a binary string jpeg file')
		}
		var blockLength = imgData.charCodeAt(4)*256 + imgData.charCodeAt(5);
		var i = 4, len = imgData.length;
		while ( i < len ) {
			i += blockLength;
			if (imgData.charCodeAt(i) !== 0xff) {
				throw new Error('getJpegSize could not find the size of the image');
			}
			if (imgData.charCodeAt(i+1) === 0xc0 || //(SOF) Huffman  - Baseline DCT
			    imgData.charCodeAt(i+1) === 0xc1 || //(SOF) Huffman  - Extended sequential DCT
			    imgData.charCodeAt(i+1) === 0xc2 || // Progressive DCT (SOF2)
			    imgData.charCodeAt(i+1) === 0xc3 || // Spatial (sequential) lossless (SOF3)
			    imgData.charCodeAt(i+1) === 0xc4 || // Differential sequential DCT (SOF5)
			    imgData.charCodeAt(i+1) === 0xc5 || // Differential progressive DCT (SOF6)
			    imgData.charCodeAt(i+1) === 0xc6 || // Differential spatial (SOF7)
			    imgData.charCodeAt(i+1) === 0xc7) {
				height = imgData.charCodeAt(i+5)*256 + imgData.charCodeAt(i+6);
				width = imgData.charCodeAt(i+7)*256 + imgData.charCodeAt(i+8);
                numcomponents = imgData.charCodeAt(i+9);
				return [width, height, numcomponents];
			} else {
				i += 2;
				blockLength = imgData.charCodeAt(i)*256 + imgData.charCodeAt(i+1)
			}
		}
	}
	, getJpegSizeFromBytes = function(data) {

		var hdr = (data[0] << 8) | data[1];

		if(hdr !== 0xFFD8)
			throw new Error('Supplied data is not a JPEG');

		var len = data.length,
			block = (data[4] << 8) + data[5],
			pos = 4,
			bytes, width, height, numcomponents;

		while(pos < len) {
			pos += block;
			bytes = readBytes(data, pos);
			block = (bytes[2] << 8) + bytes[3];
			if((bytes[1] === 0xC0 || bytes[1] === 0xC2) && bytes[0] === 0xFF && block > 7) {
				bytes = readBytes(data, pos + 5);
				width = (bytes[2] << 8) + bytes[3];
				height = (bytes[0] << 8) + bytes[1];
                numcomponents = bytes[4];
				return {width:width, height:height, numcomponents: numcomponents};
			}

			pos+=2;
		}

		throw new Error('getJpegSizeFromBytes could not find the size of the image');
	}
	, readBytes = function(data, offset) {
		return data.subarray(offset, offset+ 5);
	};

	jsPDFAPI.processJPEG = function(data, index, alias, compression, dataAsBinaryString) {
		'use strict'
		var colorSpace = this.color_spaces.DEVICE_RGB,
			filter = this.decode.DCT_DECODE,
			bpc = 8,
			dims;

		if(this.isString(data)) {
			dims = getJpegSize(data);
			return this.createImageInfo(data, dims[0], dims[1], dims[3] == 1 ? this.color_spaces.DEVICE_GRAY:colorSpace, bpc, filter, index, alias);
		}

		if(this.isArrayBuffer(data))
			data = new Uint8Array(data);

		if(this.isArrayBufferView(data)) {

			dims = getJpegSizeFromBytes(data);

			// if we already have a stored binary string rep use that
			data = dataAsBinaryString || this.arrayBufferToBinaryString(data);

			return this.createImageInfo(data, dims.width, dims.height, dims.numcomponents == 1 ? this.color_spaces.DEVICE_GRAY:colorSpace, bpc, filter, index, alias);
		}

		return null;
	};

	jsPDFAPI.processJPG = function(/*data, index, alias, compression, dataAsBinaryString*/) {
		return this.processJPEG.apply(this, arguments);
	}

})(jsPDF.API);
```

  

Documentation generated by JSDoc 3.4.2 on Sun Oct 09 2016 11:08:27 GMT+0100 (BST) using the docdash theme.
